# Outcomes of laparoscopic appendectomy of a normally appearing appendix in children with suspected acute appendicitis: a tertiary care center experience

**DOI:** 10.1186/s12887-025-05630-8

**Published:** 2025-05-06

**Authors:** Wael Abosena, Mohamed Ahmed Elghazeery, Hisham Almohamady Almetaher, Ahmed Mostafa Aboelyazeed

**Affiliations:** Pediatric Surgery Department, Tanta University Hospital, Tanta, Egypt

**Keywords:** Appendicitis, Laparoscopic appendectomy, Normal appendix, Pediatric

## Abstract

**Background:**

There is a lack of consensus among pediatric surgeons regarding the optimal management of a macroscopically normal appendix encountered during laparoscopy in children with suspected acute appendicitis. We hereby present our experience with laparoscopic appendectomy for macroscopically normal appendix in children with suspected acute appendicitis, evaluating its efficacy, safety, and postoperative histopathological findings of the excised specimens.

**Methods:**

This retrospective study was conducted on 221 children with acute right lower quadrant abdominal (RLQA) pain and had a high clinical suspicion of acute appendicitis between January 2018 and January 2022. Among these, 38 patients were found to have a macroscopically normal appendix during surgery. All patients underwent appendectomy, and the excised specimens were sent for histopathological examination.

**Results:**

Following laparoscopic appendectomy for macroscopically normal appendices in 38 children with suspected acute appendicitis, histopathological examination revealed pathological changes in 32 specimens (84%). Catarrhal appendicitis was identified in 26 cases (68%), fecalith obstruction in 5 cases (13%), and phlegmonous appendicitis in 1 case (3%), while only 6 specimens (16%) were histologically normal. The mean operative time was 51 min, with no conversions to open surgery. Postoperative complications occurred in 2 patients (5%), including one case of hyperpyrexia and one wound infection, both managed conservatively. The average hospital stay was 23.9 h, with 89% of patients discharged within 24 h. Postoperatively, all patients had complete RLQA pain resolution, with no complications or symptom recurrence during the three-month follow-up.

**Conclusion:**

Our study supports appendectomy in pediatric patients undergoing laparoscopy for suspected acute appendicitis when no other pathology is identified, even if the appendix appears normal. This approach is justified with low morbidity, short hospital stays, symptom resolution without recurrence and only 16% of normal-looking appendices confirmed as histologically normal. However, larger multi-centre studies are needed to validate and standardize this practice.

## Introduction

Acute appendicitis represents the most frequent reason for abdominal surgery in children [1]. Before the advent of laparoscopic surgery, surgeons often removed the appendix even if it appeared normal. This practice aimed to prevent diagnostic confusion in the future, as an appendix scar could be misleading. Consequently, it was common to remove a histologically normal appendix, a procedure referred to as a negative appendectomy. Reported rates of negative appendectomy in the literature range from 1.8 to 46%, with a higher incidence observed in the pediatric population [2–4]. While a negative appendectomy may seem benign, approximately 6% of patients experience complications afterwards [5].

A macroscopically normal appendix discovered during laparoscopy for right iliac fossa pain poses a clinical dilemma. There is no universally accepted protocol for managing such cases, and the long-term consequences of leaving the appendix in situ remain unclear [6]. Removing the appendix in these situations eliminates the risk of future exploratory laparoscopy for suspected appendicitis. Additionally, it prevents potential complications, such as progressive infection, inflammation, or perforation, in cases where microscopic appendicitis is present [7].

Alternatively, many argue that performing an appendicectomy on a macroscopically normal appendix may be unwarranted [8–10]. Removing a normal appendix exposes patients to potential complications, including surgical site infections, intra-abdominal abscesses, prolonged ileus, enterocutaneous fistulas, and small bowel obstruction [6]. Weighing the risk of needing a future repeat laparoscopy for appendicitis against conducting a potentially avoidable procedure during the initial surgery is crucial [11].

This study aims to evaluate the efficacy and safety of laparoscopic appendectomy for macroscopically normal appendices in children with suspected acute appendicitis, focusing on the correlation between gross features and histopathological findings of the excised specimens.

### Patients and methods

This retrospective study included 221 children (0–18 years) with acute right lower quadrant abdominal (RLQA) pain and had a high clinical suspicion of acute appendicitis admitted between January 2018 and January 2022. Ethical approval was obtained from the institution’s Research Ethics Committee. Among these patients, 38 were identified as having a macroscopically normal appendix during surgery. All participants underwent clinical examinations, blood tests (including total and differential leukocyte counts and C-reactive protein levels), urine analysis, and pelvic-abdominal ultrasound. No pediatric patients in our study required CT imaging. Patients with a history of prior lower abdominal surgeries, complicated appendicitis, or laparoscopies for known or suspected gynaecological pathologies—such as ovarian cysts, ovarian torsion, or pelvic inflammatory disease—were excluded from the study. A macroscopically normal appendix was defined as the absence of hyperemia, wall thickening, fibrin deposits, or peri-appendicular fluid.

All surgeries were performed as laparoscopic appendectomies. The specimen was retrieved via the umbilical port. All removed specimens were sent postoperatively for histopathological analysis to compare with the intraoperative gross findings without any information regarding the surgeon’s intraoperative assessment.

Histological findings were classified as positive if inflammation was detected within the appendix or its mucosal lining (catarrhal appendicitis) or if polymorphonuclear cell infiltration was observed throughout the entire thickness of the appendix wall without necrosis (phlegmonous appendicitis). Findings were considered negative in the absence of any signs of inflammation. Follow-up appointments were scheduled for all patients at 2 weeks and 3 months postoperatively.

## Results

A total of 38 patients with a macroscopically normal appendix underwent laparoscopic appendectomy. The patients’ ages ranged from 6 to 18 years, with a median age of 10 years. The cohort included 21 males (55%) and 17 females (45%). The mean operative time was 51 min (range: 25–80 min), and no cases required conversion to open surgery (Table 1).

**Table 1:** Patients’ demographics and clinical outcomes

Patients number	38
Patients sex	21 male (55%): 17 female (45%)
Median age (range)	10 (6–18 years)
Operative time (min)	51 min (25–80)
Average hospital stay length (hours)	23.9 (16–72)
Morbidity (N, %)	2 (5%)
Mortality	0

Of the 38 macroscopically normal appendices removed, histopathological examination confirmed that only 6 specimens (16%) were truly normal (non-inflamed). The remaining 32 specimens showed varying degrees of pathology. The pathologist identified catarrhal appendicitis in 26 patients (68%), characterized by luminal inflammation and mucosal erosions. In 5 patients (13%), a fecalith obstructing the lumen of the appendix with no inflammation was observed. Only one patient (3%) was found to have phlegmonous appendicitis (Table 2).

**Table 2:** Histopathology results

Histological diagnosis	Number (%)
Catarrhal appendix	26 (68%)
Fecalith appendix with no-inflammation	5 (13%)
Phlegmonous appendix	1 (3%)
Non-inflamed appendix	6 (16%)

Two postoperative complications (5%) were recorded: one case of hyperpyrexia resolved with antibiotics and one umbilical port-site infection managed with dressings and antibiotics without readmission. The average length of postoperative hospital stay was 23.9 h (range: 16–72 h) (Table 1).

Postoperative oral feeding began upon recovery, starting with sips of water and advancing as tolerated. Most patients (89%) were discharged within 24 h. Three patients (8%) had delayed feeding tolerance and were discharged within 48 h, while one (3%) developed postoperative fever and was discharged after 72 h. Postoperatively, all patients experienced significant resolution of RLQA pain, with no post-appendectomy complications or recurrence of symptoms observed during the follow-up period.

## Discussion

Although laparoscopy is increasingly used as a diagnostic tool for various acute abdominal conditions, the management of a normal appendix discovered during laparoscopy for right iliac fossa pain in pediatric patients remains a subject of debate. This is partly due to inconsistent findings in the literature, which offer limited conclusive guidance [12, 13]. Nevertheless, A review of practices across 12 pediatric emergency departments in Canada highlights the variability in managing pediatric appendicitis. The study revealed significant differences in all key aspects of care, including imaging modalities, time to surgery, surgical techniques, and antibiotic administration across the centers [14].

A recent survey conducted by Logie et al. reported that all participating surgeons from the Canadian Association of Pediatric Surgeons (CAPS) agreed on removing a normal-appearing appendix during laparoscopy for suspected acute appendicitis. The primary reasons cited included the potential for microscopic appendicitis (72.2%), the desire to prevent future diagnostic uncertainty (51.9%), and considerations related to patient preference or consent discussions (38.9%) [13]. Nearly all the surveyed surgeons (53 out of 54, 98.1%) reported having performed a negative appendectomy. Although the morbidity associated with negative appendectomy is not negligible, estimated at 6% [5], it is often justified by the perception that it is a relatively low-risk procedure with no significant added complications compared to an uncomplicated appendectomy [13, 15].

Phillips et al. recommend removing macroscopically normal appendices during laparoscopy for right iliac fossa pain if no other cause is identified, as macroscopic evaluation lacks diagnostic accuracy compared to histology and does not significantly increase operative time or port-site infection rates [16]. Similarly, Champault et al. report that laparoscopy has a 92% accuracy rate in diagnosing appendicitis, highlighting that some level of error persists even with direct visualization [17]. Ekeh et al. additionally support the removal of a normal-appearing appendix during laparoscopy when no alternate pathology is found [18]. Fadgyas et al. reported a moderate agreement between intraoperative surgical assessment and histopathological findings in pediatric appendicitis, emphasizing that intraoperative evaluation alone is not always reliable​. Their study found that when surgeons underestimated the severity of appendicitis, patients experienced longer hospital stays and higher complication rates, reinforcing the limitations of macroscopic assessment. Additionally, they reported that complicated appendicitis cases are generally distinguishable intraoperatively, but discrepancies still occur, particularly in less severe cases. These findings further support the removal of a macroscopically normal appendix during laparoscopy if no other pathology is identified, as the histological examination may reveal underlying appendiceal disease not apparent on visual inspection [19].

Conversely, some surgeons advocate leaving a macroscopically normal appendix in situ during laparoscopy, arguing that appendectomy in such cases is unnecessary and may expose patients to avoidable complications [8, 9, 11]. Pátková et al. conducted a long-term follow-up study (19–26 years) using registry data from the first two randomized clinical trials in Sweden comparing antibiotics versus surgery for acute appendicitis. While initial nonoperative treatment was successful in most cases, recurrence occurred in up to 36.8% within the first year, and appendectomies continued even decades later. By the end of follow-up, 60% of patients had avoided surgery, but they were more likely to seek medical care for abdominal pain compared to those who underwent early appendectomy. These findings concluded that nonoperative management can be a long-term option for some patients, but recurrence remains a significant risk, reinforcing appendectomy as the definitive treatment [20]. On the other hand, Emerging evidence suggests the appendix is not vestigial but a vital organ linking the immune system, nervous system, and gut microbiota. It serves as a secondary lymphoid organ and a key site for IgA immunoglobulin production [21].

In the current study, 38 of 221 patients (17%) who underwent laparoscopic appendectomy for acute right lower quadrant abdominal pain had a macroscopically normal appendix. Our findings are close to those of Tartaglia et al., Papageorgopoulou et al., and Dingemann et al., who reported rates of macroscopically normal appendices in 12%, 16.6%, and 11% of laparoscopic explorations for suspected appendicitis, respectively [9, 12, 22]. However, only 16% of macroscopically normal appendices in our study were confirmed to be truly normal upon histological examination. This rate aligns with data from other studies, which reported normal histological findings in 24% and 20% of cases, respectively [12, 22]. In our research, most of the macroscopically normal appendices (68%) were found to have catarrhal inflammation. This is consistent with the findings of Tartaglia et al., who reported catarrhal inflammation in 66% of macroscopically normal appendices [12]. Compared to studies focusing on patients undergoing diagnostic laparoscopy without appendectomy for acute right lower quadrant abdominal pain, appendectomized patients had a similar morbidity rate (5% vs. 4.5%) but shorter hospital stays (1 day vs. 3 days) [8, 21].

Microscopic appendicitis is a subject of debate, with its clinical importance and progression being increasingly questioned. Some researchers suggest that manipulating the appendix during procedures may cause inflammation, potentially affecting pathology results. Additionally, no clear explanation exists for how mucosal inflammation, confined to the inner lining and not extending to the serosa, could result in right lower quadrant pain [23–25]. Some experts view microscopic appendicitis as a distinct condition that often resolves on its own [26]. In a survey by Logie et al., 52% of participants were uncertain about its significance, while 39% believed it could cause right lower quadrant pain, and 9% disagreed.

Additionally, 50% agreed that microscopic appendicitis does not progress to acute appendicitis, 4% believed it could, and 46% were unsure [13]. There is a lack of clear evidence to assist pediatric surgeons in making decisions when they encounter a macroscopically normal appendix during laparoscopy for suspected acute appendicitis. Although some studies advocate for removing a normal-appearing appendix, the issue remains debated, and making a decision is challenging.

Our study is limited by its retrospective design and relatively small number of patients, which may affect generalizability. However, this sample represents a clinically meaningful number given the rarity of this pathology. Additionally, we did not assess the Alvarado score or other preoperative scoring systems or correlate clinical, laboratory, and imaging findings with histopathological outcomes, as this was beyond our study’s scope. Instead, we aimed to evaluate postoperative histological findings and clinical outcomes to better understand the fate of macroscopically normal appendices. Despite these limitations, our findings provide valuable insights, and future multi-center prospective studies are needed to further refine surgical decision-making.

In conclusion, our study supports performing an appendectomy in pediatric patients undergoing laparoscopy for suspected acute appendicitis, even when the appendix appears normal on visual inspection, as long as no other clear pathology explains the patient’s symptoms. This approach is reasonable and justified, given that all patients experienced significant resolution of RLQA pain, with no symptom recurrence during follow-up. Additionally, the procedure is associated with low morbidity, a shorter hospital stay, and the finding that only 16% of macroscopically normal appendices were histologically normal. However, extensive multi-center studies are urgently needed to strengthen the evidence and establish standardized guidelines for this practice.

## Data Availability

The data supporting the findings of this study are available from the corresponding author upon reasonable request.
